# Adult Langerhans Cell Histiocytosis with Hepatic and Pulmonary Involvement

**DOI:** 10.1155/2015/536328

**Published:** 2015-04-21

**Authors:** Bruno Araujo, Francisco Costa, Joanne Lopes, Ricardo Castro

**Affiliations:** ^1^Radiology Department, Centro Hospitalar de São João, Alameda Professor Hernâni Monteiro, 4200–319 Porto, Portugal; ^2^Pathology Department, Centro Hospitalar de São João, Alameda Professor Hernâni Monteiro, 4200–319 Porto, Portugal

## Abstract

Langerhans cell histiocytosis (LCH) is a rare proliferative disorder of Langerhans cells of unknown etiology. It can involve multiple organ systems with different clinical presentation, which complicates the diagnosis. It can range from isolated to multisystem disease with different prognosis. Although common among children, liver involvement is relatively rare in adults and frequently overlooked. Natural history of liver LCH fits into two stages: an early stage with infiltration by histiocytes and a late stage with sclerosis of the biliary tree. Pulmonary findings are more common and include multiple nodules in different stages of cavitation, predominantly in the upper lobes. We present a case of adult LCH with pulmonary and biopsy proven liver involvement with resolution of the hepatic findings after treatment.

## 1. Introduction

Langerhans cell histiocytosis (LCH) is a rare disease involving clonal proliferation and migration of dendritic antigen-presenting histiocytes. The typical form is more common in females (2/1.5) and usually affects children between 1 and 3 years of age [1]. Among adults the incidence is ten times less with a wide range of ages affected with a mean in the thirties and only 10% over 55 [2].

LCH can be stratified in two major categories: “single-system” or “multisystem.” Single-system is further subdivided into single site (unifocal bone, skin) and multiple site (multifocal bone, skin, or lymph nodes). Multisystem, defined as involvement of two or more organs with or without dysfunction, is subdivided into “low-risk” and “high-risk” groups. Absence of “risk” organs (liver, lungs, spleen, and hematopoietic system) involvement characterizes “low-risk” patients (approx. 20%) and is associated with a good prognosis. “High-risk” patients (80%) have at least one risk organ involved and a high mortality rate [3].

Frequency of liver involvement is known to be high (19–60%) in children and bears a poor prognosis [4, 5]. Among adults, liver involvement is rare and is poorly understood, though it is an important cause of morbidity and mortality.

## 2. Case Report

A 52-year-old male was referred to our hospital for cough with recent mild dyspnea and pleuritic chest pain. He had a personal history of tobacco (36 pack-year) and alcohol consumption (approx. 50 g/day). The physical examination showed an enlarged liver. No cutaneous lesions were found.

Initial laboratory tests showed abnormal liver enzyme tests with elevated serum aspartate aminotransferase (AST) (90 U/L) and alanine aminotransferase (ALT) (89 U/L). Serum gamma-glutamyl transpeptidase (GGT) values were mildly elevated (63 U/L) as well as alkaline phosphatase values (128 U/L). Albumin and total bilirubin levels were normal (46 g/L and 3.3 mg/L, resp.). Complete blood count was normal.

**Figure 1:**
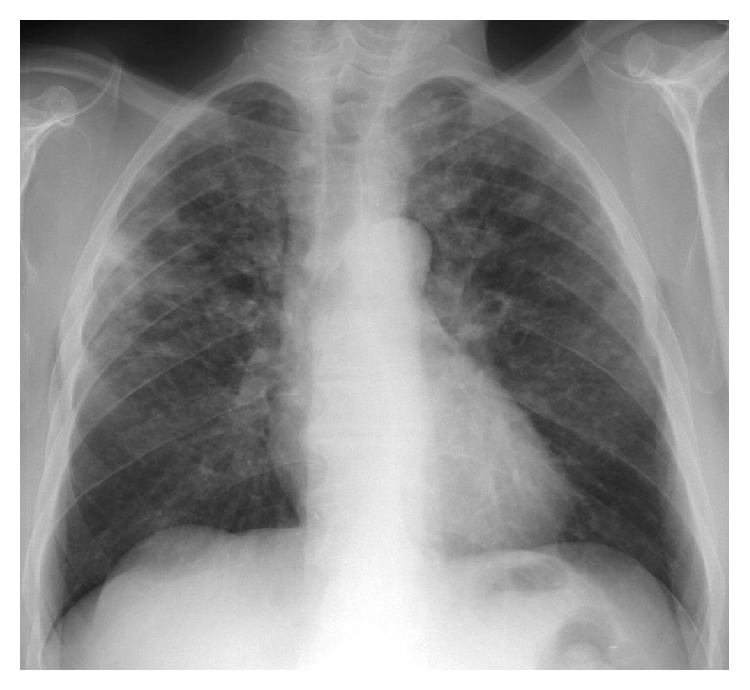
Thoracic X-ray: multiple pulmonary nodules are identified, with upper lobe predominance. Note that some nodules show cavitation.

**Figure 2:**
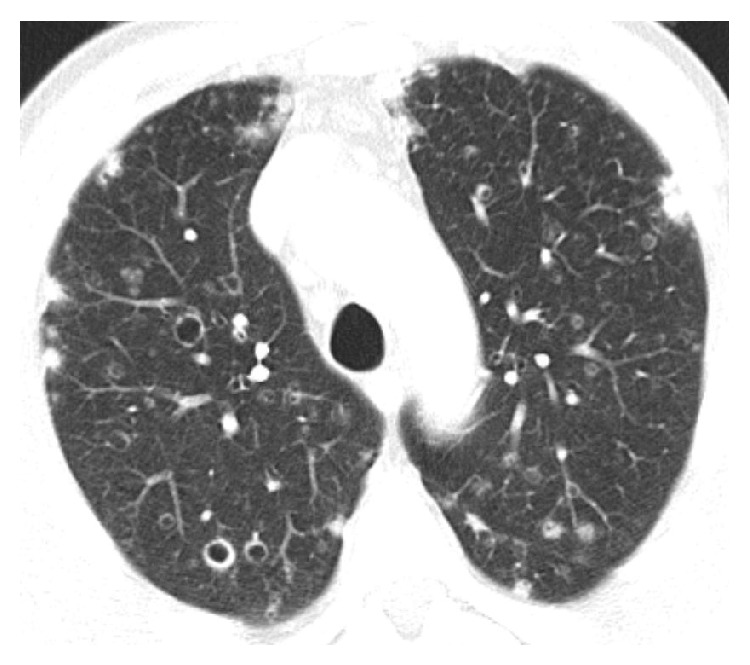
Axial thoracic CT (lung window): multiple bilateral pulmonary nodules are identified in different stages of cavitation, a typical finding of pulmonary LCH.

**Figure 3:**
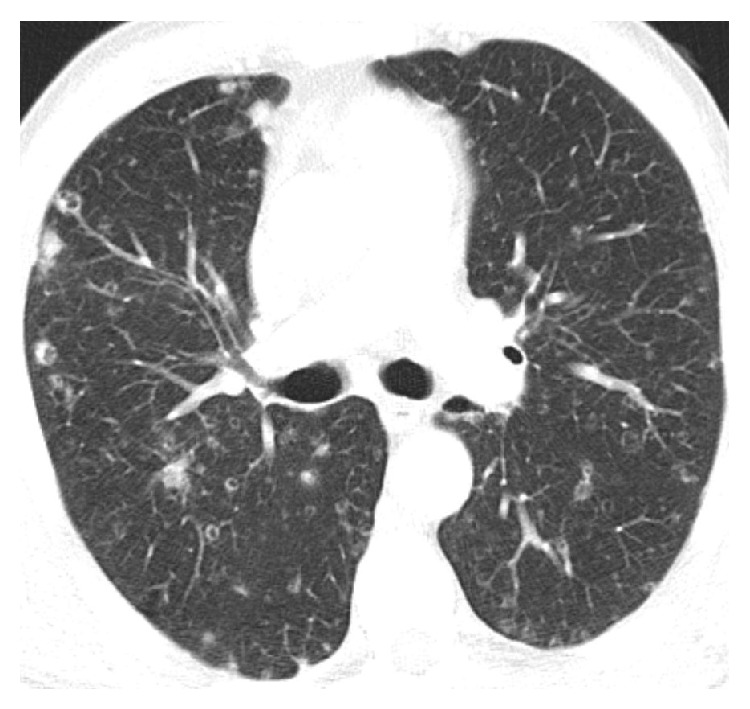
Axial thoracic CT image (lung window) in a lower thoracic CT slice: less nodules are identified. Note the normal appearance of the lung parenchyma other than the nodules.

**Figure 4:**
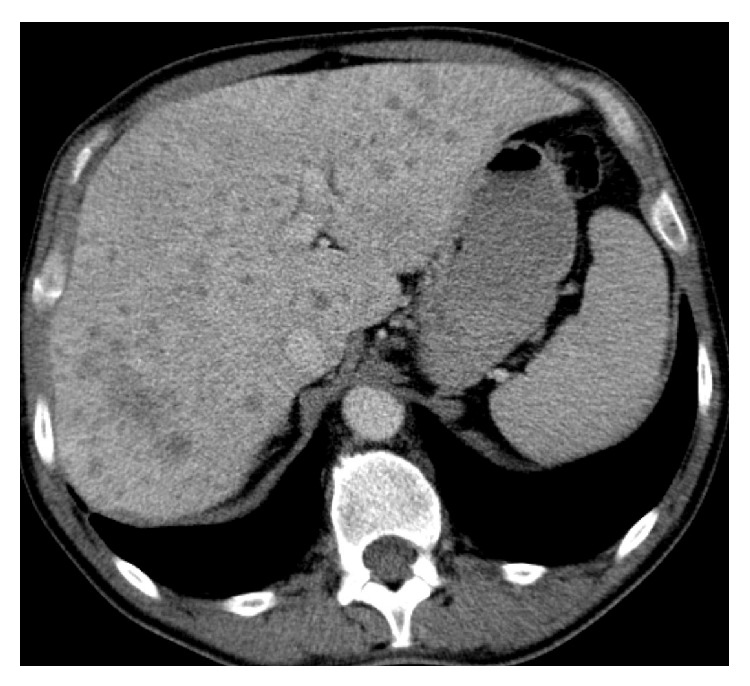
Axial contrast-enhanced abdominal CT: the patient presented increased liver dimension (hepatomegaly) in a biopsy proven liver acute involvement of LCH. Multiple hypodense nodules are identified, some of which confluent. No bile duct dilatation is present.

**Figure 5:**
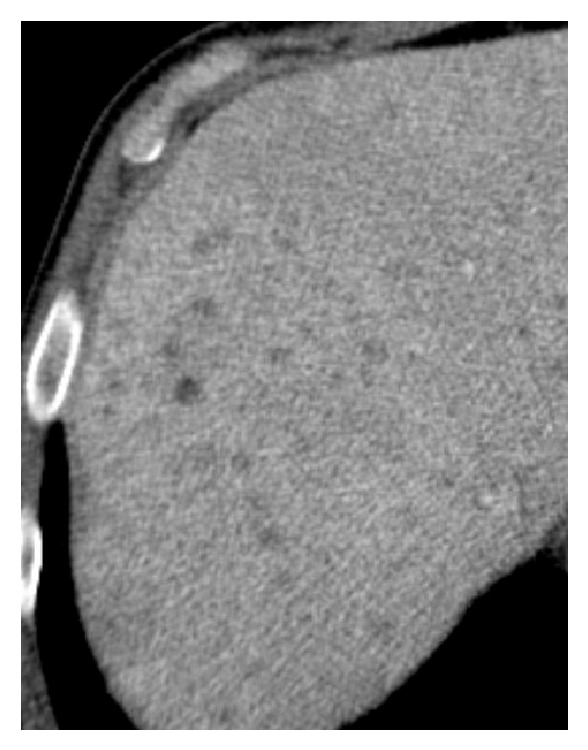
Magnification of axial contrast-enhanced abdominal CT: liver nodules can be best appreciated, some confluent. Note the hypodense area (fat attenuation values) of one of the nodules, indicating xanthomatous type lesion.

A thoracic X-ray was performed demonstrating multiple nodules with upper predominance, some of which appear cavitated (Figure 1). The thoracic and abdominal enhanced computed tomography (CT) confirmed the presence of multiple infracentimetric centrilobular nodules, some of them cavitated, and cysts (Figures 2 and 3). The surrounding pulmonary parenchyma was normal. These findings predominated in the superior lobes with sparing of the most inferior segments. Hepatomegaly was also present with multiple hypodense hepatic nodules identified, measuring up to 15 mm, some of them confluent. Bile ducts dilatation was not present. No other abdominal abnormalities were found (Figures 4 and 5).

**Figure 6:**
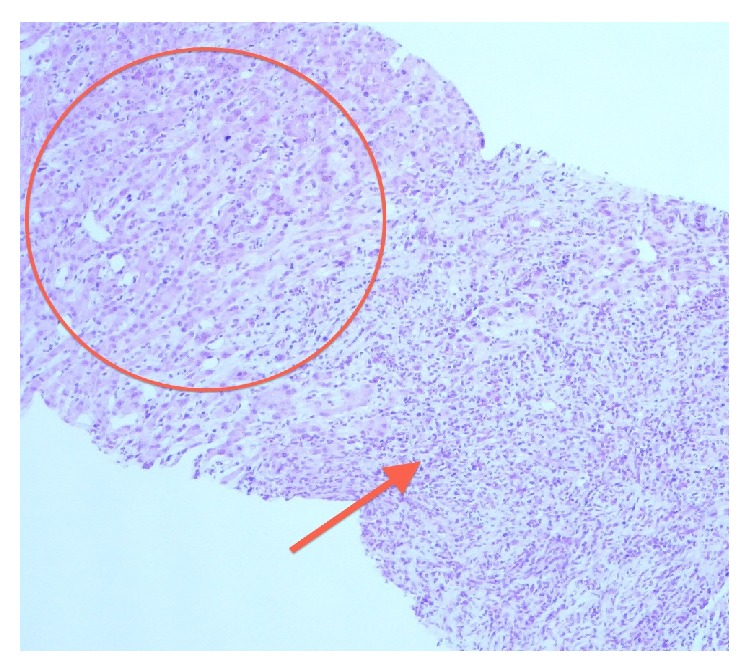
Photomicrographs (hematoxylin-eosin stain 100x magnification) of the liver biopsy specimens: normal hepatic parenchyma can be identified (circle) with cords of hepatocytes and normal portal spaces. Lymphocytic infiltration of the liver parenchyma can be identified (arrow) with some multinucleated cells (Langerhans cell).

**Figure 7:**
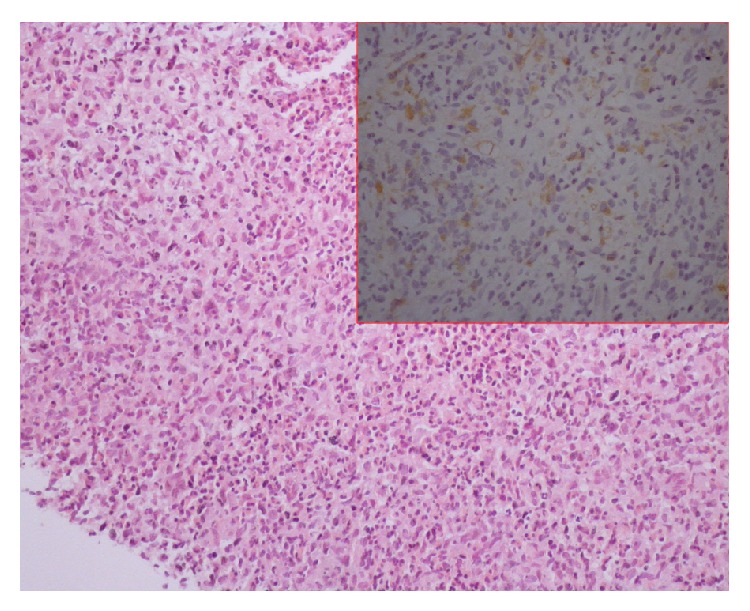
Photomicrographs (hematoxylin-eosin (H-E) stain 200x magnification and CD1a immunolabeling) of the liver biopsy specimens: lymphocytic infiltrate is identified with multiple Langerhans cell intermixed that stain positive to CD1a, compatible with liver involvement of LCH.

These findings suggested Langerhans' cell histiocytosis with pulmonary and hepatic involvement. A liver biopsy was performed demonstrating infiltration of large cells with irregular nuclei that positively stained to CD1a and S100, compatible with early liver involvement of LCH (Figures 6 and 7).

**Figure 8:**
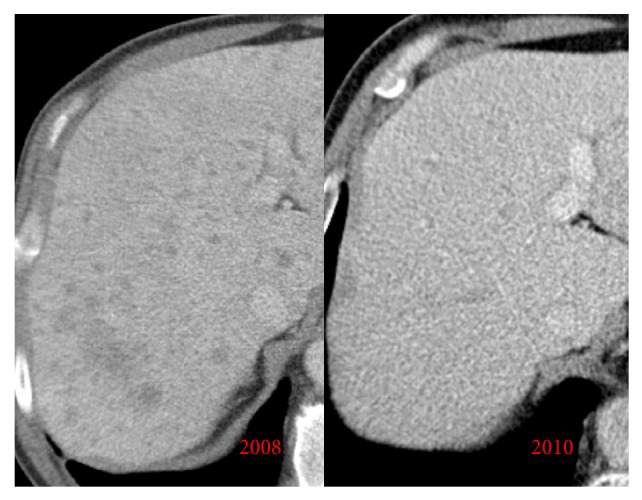
Axial contrast-enhanced abdominal CT images: comparison of CT abdominal images from 2008 and 2010 after chemotherapy, demonstrating resolution of the liver nodules, common in acute liver involvement of LCH.

Treatment with vinblastine and prednisone was initiated with a favorable response. No liver nodules were found in follow-up CTs (Figure 8).

## 3. Discussion

Liver involvement in children with multisystem LHC is known to be high with reported incidence values from 19% to 60%, bearing a poor prognosis [6]. Early liver involvement often responds to chemotherapy/bone marrow grafting strategy [7, 8].

However, in adults, liver involvement in LHC is poorly recognized and frequently overlooked with an incidence reported values between 16% and 27%, although 87% occurred in multisystemic LHC [9, 10].

A study described two distinct forms of LCH liver involvement: first, an early LCH liver involvement secondary to Langerhans' cell infiltration of the liver, usually presenting with hepatomegaly and liver nodules, and often responsive to immunosuppressive/chemotherapy treatment. Cholestasis, if present, is mild. And a second form is described as late LCH liver involvement which is associated with chronic fibrosis centered on bile ducts with little or no histiocytic infiltration, progressing to sclerosing cholangitis [10]. The presence of CD1a-positive cells aids in the distinction between primary sclerosing cholangitis, which may be difficult if the biopsy is done very late with only fibrotic lesions [11].

In case of liver nodules, the differential diagnosis of LCH liver involvement is difficult with other diagnoses as primary or secondary tumors of the liver. The possible association between LCH and malignant tumors can make it even more complicated [12, 13]. The discovery of other localizations of LCH, as the lung in the case presented, and the absence of cancer help reaching the diagnosis of hepatobiliary LCH.

The prognosis of LCH is worst if liver involvement is present with a fatality rate of 30% (versus 10% without liver involvement). Early LCH liver involvement with either nodules or hepatomegaly secondary to histiocytes infiltration is usually responsive to treatment as found in our case with complete resolution of the liver nodules. Late LCH liver involvement with sclerosing cholangitis, cirrhosis, or liver insufficiency is difficult to treat with very low and partial response to treatment. In the end, the treatment that can be proposed is liver transplantation [14].

## 4. Conclusion

LCH liver involvement is uncommon in adults and should be thought of not only in “multisystem” LCH but also in “single-system” LCH and can even be the first manifestation of the disease. Usually under diagnosed, is very important to identify liver involvement it at an early reversible stage, allowing a better prognosis. Liver palpation, liver function test, and imaging are key tools to reach the diagnosis. Liver biopsy is still the cornerstone of the diagnosis enabling specific immunohistochemistry studies and liver disease staging.

Reaching an accurate diagnosis as soon as possible allows earlier treatment of liver disease with a significant improvement in prognosis.
